# Dietary *Artemisia arborescens* Supplementation Effects on Growth, Oxidative Status, and Immunity of Gilthead Seabream (*Sparus aurata* L.)

**DOI:** 10.3390/ani14081161

**Published:** 2024-04-11

**Authors:** Odysseas-Panagiotis Tzortzatos, Dimitra K. Toubanaki, Markos N. Kolygas, Yannis Kotzamanis, Efstratios Roussos, Vasileios Bakopoulos, Achilleas Chatzopoulos, Fotini Athanassopoulou, Evdokia Karagouni

**Affiliations:** 1 Immunology of Infection Group, Department of Microbiology, Hellenic Pasteur Institute, 11521 Athens, Greece; ptzortzatos@pasteur.gr (O.-P.T.); dtouban@pasteur.gr (D.K.T.); 2 Laboratory of Ichthyology & Fish Pathology, Faculty of Veterinary Medicine, University of Thessaly, 43100 Karditsa, Greece; kolygasmarkos@gmail.com (M.N.K.); eathan@vet.uth.gr (F.A.); 3 Institute of Marine Biology, Biotechnology and Aquaculture, Hellenic Centre for Marine Research (HCMR), 19013 Athens, Greece; jokotz@hcmr.gr (Y.K.); e.roussos@hcmr.gr (E.R.); 4 Department of Marine Sciences, School of the Environment, University of the Aegean, University Hill, Lesvos, 81100 Mytilene, Greece; v.bakopoulos@aegean.gr; 5 Skaloma Fishery, 46300 Sagiada, Greece; chatzopoul@gmail.com

**Keywords:** seabream, *Sparus aurata*, *Artemisia arborescens*, essential oil, feed additive, growth performance, immune response, oxidative stress

## Abstract

**Simple Summary:**

Aquaculture is predicted to play a crucial role in providing nutritional and healthy food for people worldwide. Under the Blue Transformation concept, aquaculture should be sustainable and resilient, minimize environmental impacts, and improve biosecurity and disease control. The use of essential oils and extracts of various species of edible and medicinal plants, herbs, and spices is considered promising as options for aqua feed additives, paving a sustainable route of fish protection using safe, eco-friendly compounds in a more cost-effective way for treatment compared to antibiotics currently used. Our aim was the evaluation of the impact of *Artemisia arborescens* dietary supplementation on growth performance, biochemical and oxidative stress, and immunological parameters on gilthead seabream (*Sparus aurata*). Our findings suggested that the use of *A. arborescens* as a feed supplement has a compromised positive effect on the above-mentioned parameters of gilthead seabream; therefore, plant-origin feed additives should be used in fish species with caution.

**Abstract:**

Fish infectious diseases are one of the main constraints of the aquaculture sector. The use of medicinal plants provides a sustainable way of protection using safe, eco-friendly compounds in a more cost-effective way of treatment, compared to antibiotics. The aim of the present study is the assessment of *Artemisia arborescens* (AA) feed-supplementation effects on gilthead seabream (*Sparus aurata*). Fish with an average initial body weight of 109.43 ± 3.81 g, were divided into two groups based on AA feed composition (A25 and A50). Following two months of ad libitum feeding, the effect of diets on fish weight and length were measured. Fish serum and mucus were analyzed for non-specific immune parameters (nitric oxide, lysozyme, myeloperoxidase, protease-/anti-protease activity, and complement), antibody responses, oxidative stress (cytochrome P450 1A1, metallothionein), and metabolism markers (total protein, alkaline phosphatase, and glucose). Expression levels of antioxidants (*sod1*, *gpx1*), cytokines (*il-1b*, *il-10*, *tfgb1*, and *tnfa*), *hepcidin*, and heat shock protein *grp75* genes were measured in spleen samples. A results analysis indicated that *A. arborescens* use as a feed supplement has a compromised positive effect on the growth performance, immune response, and blood parameters of gilthead seabream. Overall, the suitability of *A. arborescens* as an efficient food supplement for gilthead seabream health improvement was investigated, setting the basis for its application assessment in Mediterranean aquaculture.

## 1. Introduction

Aquatic food systems play a crucial role in providing people worldwide with essential protein and nutrients [1], and aquaculture is predicted to supply the majority of aquatic dietary protein by 2050 [2]. Under the Blue Transformation concept, aquaculture should be sustainable and resilient, minimize environmental impacts, and improve biosecurity and disease control with the support of technology and innovation [1]. Even though the industry is rapidly increasing, aquaculture faces challenges, including the effects of pathogens, pests, pollution, and climate change, with great environmental and economic implications. The use of chemicals for the prevention and treatment of pathogens has become a common practice; however, improper use can be dangerous for the health of consumers, workers, cultured organisms, and surrounding ecosystems [3]. More specifically, the use of antibiotics to control fish diseases could lead to accumulation in tissues, give rise to drug-resistant pathogens, and result in immune-system suppression. As an alternative, supplementation of feed with plant extracts, nutraceuticals, prebiotics, and probiotics can be used to boost fish growth and immunity [3,4]. Therefore, sustainable approaches for antibiotic-use elimination and fish health status enhancement are in high demand, especially in low- and middle-income countries (LMICs) [5,6].

Essential oils and extracts of various species of edible and medicinal plants, herbs, and spices are considered promising options as aqua feed additives since they have widely recognized antimicrobial and therapeutic effects and are biologically safe [7]. Feed supplementation with plants has been proven to increase growth and feeding efficiency and improve hematological and immune parameters in serum and mucus [6]. The antimicrobial components that are included in plant essential oils and extracts (usually in high concentrations) could reduce the risk of infections in fish and help maintain a healthy gut microbiota [6,7]. Moreover, plant essential oils and extracts have antioxidant properties and could decrease oxidative stress side-effects on fish that may be caused by environmental factors [8].

*Artemisia (A.)* is one such plant that belongs to the genus of the Asteraceae family with more than 300 species. Different *Artemisia* species exhibit neuroprotective, anti-depressant, cytotoxic, anti-malaria (artemisinin), digestive, nephro- or hepato-protective properties, and antimicrobial activities. Several classes of secondary metabolites, including terpenoids, sesquiterpenoids, flavonoids, lactones, lignans, coumarins, glycosides, caffeoylquinic acids, sterols, and polyacetylenes, have been identified in *Artemisia* species [9,10].

*Artemisia arborescens* is mainly found in the Mediterranean and Pacific North America areas [8]. A recent study on *A. arborescens*, isolated in Greece, identified medium-to-high levels of chlorogenic acid, glucosides, gallic acid derivative, quercetins, and bilactones, among other substances [11].

Recent studies have assessed the potential use of *Artemisia* extracts on fish growth and health promotion. Among these, dietary supplementation with *A. dracunculus* was studied on rainbow trout (*Oncorhynchus mykiss*) resulting in improved fish growth, blood biochemical parameters, and immune responses [12]. *A. annua* and *Artemisia absinthium* have been incorporated in common carp (*Cyprinus carpio*) feed, where it was found that these *Artemisia* species had considerable potential as natural immunostimulant and growth-promotor supplements [9,13,14]. *A. annua* effects have also been assessed on Nile tilapia (*Oreochromis niloticus*) [15] and largemouth bass (*Micropterus salmoides*) [16]. In these studies, it was also found that *A. annua* could minimize stress response and modulate innate immunity in Nile tilapia [15], enhance liver antioxidant capacity, improve intestinal morphology, and regulate intestinal flora of largemouth bass [16]. *Artemisia capillaries* has been used in red seabream (*Pagrus major*) and Japanese flounder (*Paralichthys olivaceus*) feeds, alone or as part of an herbal mix that improves fish health parameters [17,18,19]. Finally, *Artemisia afra* has been tested on *Oreochromis mossambicus* [20] and *Clarias gariepinus* [21], and it was assumed that it could be used as a dietary additive to improve disease resistance without compromising fish growth in a specific range of concentrations.

The gilthead seabream (*Sparus aurata* L.) is one of the main commercial species (in terms of value) in the Mediterranean [22] and ranks fourth in the world production of finfish in marine and coastal aquaculture [23]. It has an estimated annual production volume of 258,754 T/year by the leading six producers worldwide (i.e., Turkey, Greece, Egypt, Tunisia, Spain, and Italy). Its 2018 worldwide exports of 130,042 tons were valued at USD 653 million, and imports of 100,584 tons were valued at USD 532 million [24]. Gilthead seabream is generally considered a robust and pathogen-resilient species; however, it is highly affected by bacteria (e.g., *Tenacibaculum maritimum*), parasites (e.g., *Sparicotyle chrysophrii*, *Enterospora nucleophila*, *Enteromyxum leei*, and *Amyloodinium ocellatum*), and viruses (e.g., nervous necrosis virus; NVV) [25]. Among other pathogens, Myxobacteria are commonly affecting Greek aquaculture. On the other hand, *Artemisia arborescens* is widely spread in Greece, and its essential oil can be obtained at a low cost. Based on the above-mentioned studies of various *Artemisia* species to fish, it is anticipated that *Artemisia* feed supplements could improve fish health and growth, creating a promising area for seabream-related research. To the best of our knowledge, this is the first study of dietary *Artemisia arborescens* effects on *Sparus aurata*.

The aim of the present study was the evaluation of the impact of *Artemisia arborescens* dietary supplementation on growth performance, biochemical and oxidative stress, and immunological parameters on mucus and serum in gilthead seabream. Gene-expression levels for selected markers of oxidative stress, metabolism, and immune responses were also analyzed to assess the *A. arborescens* essential-oil benefits on fish reared in aquaculture farms.

## 2. Materials and Methods

### 2.1. Ethics Statement

The trial’s experimental protocol underwent scrutiny and received approval from the Research Ethics Committee in accordance with the Hellenic presidential decree 56/2013 (106). Accredited scientists oversaw the care, procedures, sampling, anesthesia, and euthanasia of the experimental gilthead seabream. The experimental methods adhered to the principles outlined in the prevailing European Directive (2010/63/EU) safeguarding animals utilized in scientific research. Any by-products derived from experimental vertebrate animals were managed in compliance with Regulation (EC) No 1069/2009.

### 2.2. Experimental Diets

For the purpose of this experiment, a basal diet was formulated using Allix 3 formulation software (A-Systems, Paris, France) and served as a control diet (cntr), following the current industrial standards to meet the nutritional requirements of the gilthead seabream. The control diet was produced by cooking–extrusion employing a lab-scale twin-screw extruder (CLEXTRAL, Firminy, France), with an extrusion temperature of less than 100 °C at the HCMR’s feed production facilities in Anavyssos, Attiki, Greece. Two other identical diets (A25 and A50) were produced under the same conditions, and they were supplemented with 0.25 and 0.5% *Artemisia arborescens* (AA) essential oil (Paggaioils, Greece), respectively, using a lab-scale vacuum coater (PEGASUS^®^ Dinnissen BV, Sevenum, Netherlands) after mixing with a quantity of fish oil and heating at 40 °C. The ingredients and chemical composition of the diets are presented in Table 1.

### 2.3. Animals and Experimental Design

A growth trial was conducted in the experimental facilities of the University of Thessaly in Greece, with three independent replicates. A total of 297 gilthead seabream (*Sparus aurata*) of both sexes, with an average initial body weight of 109.43 ± 3.81 g, were individually weighed and then randomly allocated into nine tanks, each with a capacity of 850 lt (with 33 individuals per tank). Prior to the feeding experiments, the experimental fish underwent an adaptation phase for a 15-day period, during which they were provided with a control diet. Three triplicate experimental groups were established: Group A served as the control and received the control diet (cntr); Group B was fed with *Artemisia* at a concentration of 0.25% *w*/*w* of feed (A25); and Group C was fed with *Artemisia* at a concentration of 0.5% *w*/*w* of feed (A50). Each diet was administered twice daily at 09:00 h and 15:00 h, with a feeding rate of 1.5 (FR), seven days a week, for a consecutive period of eight weeks.

On the starting day (day 0), each member of the initial population underwent individual weighing. Prior to weighing, the fish were anesthetized using Benzocaine (200 mg mL^−1^, veterinary license: 79099/01-11-2016). Each tank was equipped with a recirculating water system set at a salinity of 35 parts per thousand (ppt), with a relative renewal rate of 53 liters per hour. Furthermore, aeration was provided to ensure oxygen saturation levels remained above 75%. Throughout the acclimatization and experimental periods, the water temperature was maintained at an average of 23.7 °C ± 2.9, adhering to the natural photoperiod cycle, and the pH ranged between 7.9 and 8.1. The filtration process encompassed mechanical, biological, and chemical components.

All fish underwent a clinical examination to ensure they were in good health, checking for any signs of infestation, skeletal or soft tissue deformities, ocular injuries, or wounds. Prior to the experiment, measures were taken to prevent overcrowding and ensure equal growth and feed-intake potential for all experimental fish.

Throughout the trial, all experimental groups were exposed to similar natural environmental conditions, including photoperiod, water temperature, water chemical parameters, and cohabitation potential. The exact amount of feed given to each tank (feed intake) was recorded daily. Feeds were distributed over the entire water surface to be easily accessible to all the fish. Daily inspections were carried out for all groups to monitor the mortality rates.

At the end of the feeding trial, all fish were counted to calculate survival. Then, ten fish were randomly sampled from each tank, anesthetized, and weighed individually. Skin-mucus samples were collected by gently rubbing the lateral surfaces of the seabream specimens with a cell scraper, taking care not to contaminate the samples with blood or urogenital or intestinal excretions. Collected skin-mucus samples were diluted with an equal volume of phosphate-buffered saline (PBS: 0.14 M NaCl, 10 mM sodium phosphate, 2.7 mM KCl, and 1.7 mM potassium phosphate, pH 7.4) and stored at −20 °C before use. Blood was drawn by the caudal vein and was allowed to clot at room temperature (RT) for 30 min. Serum was isolated by centrifugation at 150× *g* for 10 min at 4 °C. Following this, spleen tissues were removed aseptically, weighted, and stored in an RNAlater (Qiagen, Hilden, Germany) at −80 °C.

### 2.4. Growth-Performance Parameters

The growth variables, somatic indices, and survival were calculated using the following formulae:

**Weight gain** (WG, %) = [(final body weight − initial body weight)/initial body weight] × 100.

**Feeding rate** (FR, %) = [daily food offered/fish biomass] × 100.

**Specific growth rate** (SGR, %/day) = [(ln final body weight − ln initial body weight)/days of experiment] × 100.

**Feed conversion ratio** (FCR) = feed intake/wet weight gain.

**Survival** (%) = (final number of fish/initial number of fish) × 100.

### 2.5. Biochemical Parameters

#### 2.5.1. Total Protein Levels

The total protein concentration in skin-mucus (1:10 dilution) and serum (1:100 dilution) samples was determined using the Protein Determination (BCA) Kit (Cat. No. 701780, Cayman Chemical, Ann Arbor, MI, USA, Rockford, IL, USA), following the manufacturer’s instructions. Serial dilutions of bovine serum albumin (Protein Determination BSA Standard, Cat. No. 704003, Cayman Chemical) were used for the standard curve. Absorbance measurements were read at 570 nm in an MRX spectrophotometer (Dynatech Laboratories, Guernsey, UK). The total protein concentration present in each sample was expressed as mg mL^−1^.

#### 2.5.2. Alkaline Phosphatase Levels

The alkaline phosphatase (ALP) concentration in skin mucus and serum samples (1:10 dilution) was measured using the Alkaline Phosphatase Colorimetric Activity Assay kit (Cat. No. 701710, Cayman Chemical), following the manufacturer’s instructions. Absorbance measurements were read at 405 nm. The ALP concentration present in each sample was calculated as follows: ALP Activity (units L^−1^)= (A_405_ × 0.15 mL)/(8.34 mM^−1^ × 20 min × 2·10^−5^ L) and expressed as units L^−1^.

#### 2.5.3. Glucose Levels

The glucose concentration in the serum samples (1:5 dilution) was measured using the Glucose Colorimetric Assay Kit (Cat. No. 10009582, Cayman Chemical), following the manufacturer’s instructions. Absorbance measurements were read at 490 nm. The glucose concentration present in each sample was expressed as mg dL^−1^.

### 2.6. Oxidative Stress Parameters

#### 2.6.1. Cytochrome P450 1A1 Levels

The cytochrome P450 1A1 concentration in the serum samples (1:50 dilution) was determined using the ELISA Fish Cytochrome P450 1A1 (CYP1A1) Kit (Cat. No. CSB-EL006395FI, Cusabio, Houston, TX, USA), following the manufacturer’s instructions. Absorbance measurements were read at 450 nm. The CYP1A1 concentration present in each sample was expressed as ng mL^−1^.

#### 2.6.2. Metallothionein Levels

The metallothionein (MT) concentration in serum samples (1:200 dilution) was determined using the ELISA Fish metallothionein Kit (Cat. No. CSB-EQ027262FI, Cusabio), following the manufacturer’s instructions. Absorbance measurements were read at 450 nm. The MT concentration present in each sample was expressed as ng mL^−1^.

### 2.7. Immunological Parameters

#### 2.7.1. Nitric Oxide Levels

Nitric oxide (NO) production was measured indirectly through the nitrite/nitrate content by the standard Griess reaction. Skin-mucus and serum samples (100 μL; 1:10 dilution) were mixed with an equal volume of Griess reagent (1% sulfanilamide and 0.1% N-1-naphthylethyleme diamine hydrochloride in 10% HCl). Serial dilutions of NaNO_2_ were used as standards. Absorbance was measured at 570 nm following a 10 min incubation at room temperature (RT). All results were expressed in µM of nitrite.

#### 2.7.2. Lysozyme Activity

Lysozyme was determined based on a turbidimetric method, with some modifications [27]. *Micrococcus lysodeikticus* (Sigma, Darmstadt, Germany) was used as a target and was suspended in 67 mM phosphate buffer (pH 6.25) to a concentration of 350 µg mL^−1^ (Ml working solution). Skin-mucus and serum samples were heat-inactivated (56 °C, 30 min) to deactivate the complement complex. Following this, 10 µL of sample serum were added to 190 µL of Ml working solution, and the absorbance was measured at 450 nm at 30 sec and 30 min. The lysozyme concentration was determined based on a 6-point standard curve (2–20 µg mL^−1^) constructed with an egg-white lysozyme standard (Sigma). The lysozyme activity for each sample was expressed as µg mL^−1^.

#### 2.7.3. Myeloperoxidase Activity

The myeloperoxidase (MPO) activity was determined as previously described [28]. Briefly, 15 μL of sample skin mucus or serum (diluted 1:5 in Hank’s Balanced Salt Solution (HBSS) without Ca^2+^ or Mg^2+^, containing 0.1% (*w*/*v*) Bovine Serum Albumin (BSA)), were mixed with 135 μL HBSS in flat-bottomed 96-well plates. Then, 50 µL of 2 mM 3,3′,5,5′-tetramethylbenzidine hydrochloride (TMB, Sigma) and 50 µL of 0.02% (*v*/*v*) H_2_O_2_ were added. The color reaction was stopped after 2 min by adding 50 μL of 0.5 M H_2_SO_4_, and the optical density was read at 450 nm. The MPO concentration was determined based on a standard curve (0.036–1 µg mL^−1^) constructed with horseradish peroxidase (Sigma). The MPO activity present in each sample was expressed as µg mL^−1^.

#### 2.7.4. Protease Activity

The protease activity was determined according to Ross et al. [29], with some modifications. Twenty μL of sample skin mucus (undiluted) or serum (1:2 dilution) were mixed with 100 μL of azocasein solution (1% (*w*/*v*)), and the mixture was incubated for 19 h at 30 °C. Following this, 100 μL of TCA (10% (*w*/*v*)) were added and incubated for 30 min (RT). The mixtures were centrifuged (400× *g*, 10 min), and 100 μL of each supernatant were transferred to a clean flat-bottom 96-well transparent microplate containing 100 μL of 1 M NaOH per well. The absorbance was measured at 450 nm. Each sample was expressed as the percentage of protease activity. For positive and negative controls, serum was replaced by trypsin (5 mg mL^−1^; 100% of protease activity) or buffer (0% activity), respectively.

#### 2.7.5. Anti-protease Activity

The anti-protease activity was determined as follows [30]: 10 μL of skin-mucus sample (undiluted) or 5 μL of sample serum (1:2 dilution) were mixed with a standard trypsin solution (20 μL; 5 mg mL^−1^, Lonza, Basel, Switzerland) and incubated for 10 min (RT). Azocasein solution (60 μL; 1% (*w*/*v*), Sigma-Aldrich, St. Louis, MO, USA) was added to the mixture and incubation continued for 1 h (RT). Following this, trichloroacetic acid (TCA) (100 μL, 10% (*w*/*v*), Applichem, Darmstadt, Germany) was added and incubated for 30 min. The mixtures were centrifuged (1900× *g*, 10 min), and 100 μL of each supernatant were transferred to a clean flat-bottom 96-well transparent microplate containing 100 μL of 1 M NaOH per well. The absorbance was measured at 450 nm. Each sample was expressed as the percentage of trypsin inhibition. The percentage inhibition of trypsin activity was calculated by comparing it with a 100% control sample, in which buffer replaced the serum, while for the negative control, buffer replaced both serum and trypsin, based on the equation % Trypsin inhibition = (OD_Trypsin_ − OD_Sample_)/OD_Trypsin_ × 100.

#### 2.7.6. Complement C3 Levels

The complement C3 concentration in the serum samples (1:100 dilution) was determined using the ELISA Fish Complement 3 Kit (Cat. No. CSB-E09727s, Cusabio, Houston, TX, USA), following the manufacturer’s instructions. Absorbance measurements were read at 450 nm. The C3 concentration present in each sample was expressed as mg mL^−1^.

#### 2.7.7. Total Immunoglobulins and Immunoglobulin M (IgM) Levels

The immunoglobulin M antibody levels were determined in fish serum samples (1:100 dilution) using the ELISA Fish Immunoglobulin M (IgM) kit (Cat. No. CSB-E12045Fh, Cusabio) following the manufacturer’s instructions. The absorbance measurements were read at 450 nm and the IgM concentration in each sample was expressed as μg mL^−1^.

The total antibody levels of all the serum samples were measured using an indirect ELISA. Briefly, 96-well plates were coated with 80 μL/well of 0.001% poly-L-lysine in a bicarbonate/carbonate buffer, pH 9.6, (incubation 1 h, RT). The plate was washed 3 times with LSWB (Low salt washing buffer (LSWB-10x): 200 mM Tris, 32.8 M NaCl, 5 mL L^−1^ Tween-20, pH 7.4), and 50 μL of seabream sera samples (1:10 dilution in 0.02 M PBS + 0.1% Tween-20) were added, followed by overnight incubation (4 °C). The plate was washed 3 times with LSWB. One hundred μL of 10% BSA in LSWB were added into each well and incubation was continued overnight (4 °C). The plate was washed with LSWB (5 times), left to soak for 5 min, and the supernatant was aspirated. Following this, 50 μL of anti-seabream IgM MAb (1:23 dilution, product FO3, Aquatic diagnostics Ltd., Scotland, UK), were added (incubation 1 h, RT). The plate was washed with LSWB (5×); 50 μL of anti-mouse IgG-biotin antibody (Sigma-Aldrich) were added (1:1000 dilution in 5% BSA in LSWB) and incubated for 45 min at RT. The plate was washed with LSWB, and 80 μL of chromogen (20 mL substrate buffer, 200 μL stock TMB, and 6.7 μL H_2_O_2_, warmed at 37 °C,) was added for 3 min. The reaction was stopped using 40 μL of 2 M H_2_SO_4_. The optical density was measured at 450 nm in a spectrophotometer (MR-96A microplate reader, MINDRAY, Shenzhen, China).

All analyses were performed in triplicate unless it is otherwise stated. All absorbance measurements were performed on the MRX spectrophotometer (Dynatech Laboratories, Guernsey, UK) unless it is otherwise specified.

### 2.8. RNA Isolation and Complementary DNA (cDNA) Synthesis

Total RNA from the spleen was extracted for eight fish from each tank. Tissue from each sample was homogenized using the TissueLyzer mechanical homogenizer (Qiagen, Hilden, Germany) with 5 mm steel beads (Qiagen) and the TRIzol Reagent solution (Invitrogen, Carlsbad, CA, USA), following the manufacturer’s instructions. RNA quantity and quality were assessed by spectrophotometry (NanoDrop 2000; Thermo Fisher Scientific, Waltham, MA, USA). Only RNA samples of high quality were used for constructing the cDNA libraries. Total RNA was used as a template to synthesize cDNA using the PrimeScript RT Reagent Kit (Takara, Shiga, Japan) according to the manufacturer’s instructions. Approximately 1 μg RNA was used as input material.

### 2.9. Gene-Expression Analysis

The gene-expression profile was analyzed to evaluate the modulation of immune-, oxidative stress- and metabolism-related genes in gilthead seabream fed with the two experimental diets: A25 and A50. Eight fish spleens were used in the gene-expression studies, namely those that had adequate total RNA with the best quality for cDNA preparation. The ribosomal protein S18 gene (*rsp18*) was employed as a reference gene, and the control group’s gene expression was used to determine the fold change for each gene in the experimental diet groups. The studied genes in the seabream spleen were related to cytokines (*il-1b*, *il-10*, *tgfb1*, *tnfa*, and *hep*), oxidative stress (*sod1*, *gpx1*), and metabolism (*grp75*). In detail, real-time PCR assays were carried out to analyze the expression patterns of the oxidative stress-, metabolism- and immune-related genes in the gilthead seabream spleen. Specific primers used for gene-expression analysis (Table S1; [31,32,33]) were designed utilizing Primer-Blast, IDT_Primer Quest, and Primer3Plus or retrieved from published studies. The qRT-PCR primers were designed according to the minimum information needed for the publication of the qRT-PCR experiments (MIQE) guidelines [34]. Before sample-quantification experiments, the specificity of each primer pair was studied using positive and negative samples.

Real-time PCR reactions were performed with KAPA SYBR FAST qPCR Kit Master Mix (KapaBiosystems, Wilmington, MA, USA) using 1 µL of a 1:2.5 dilution of cDNA. Primers for all genes were used at 10 μM. RNA sample, cDNA synthesis master mix, and ddH_2_O were used as real-time PCR internal amplification controls. The thermal conditions used were as follows: 3 min at 95 °C of pre-incubation followed by 40 cycles at 95 °C for 10 s and 60 °C for 30 s. An additional temperature-ramping step was utilized to produce melting curves from 62 to 95 °C to verify the amplification of gene-specific products on all samples. All reactions were performed in technical triplicates using a RotorGene Q PCR Detection System (Qiagen).

Quantification was done according to the comparative C_T_ method [35]. The value for each experimental condition was expressed as normalized relative expression, calculated in relation to the control group’s mean value and normalized against those of the reference gene (by its geometric average). The results were expressed as the average values for each fish. A melting-curve analysis of the amplified products validated the primers for specificity.

### 2.10. Statistical Analysis

Effects of *Artemisia arborescens* dietary supplementation on different parameters were evaluated by one-way analysis of variance (ANOVA). Tanks were considered as experimental units, and fish represented the sample units. All data were tested for normality and homogeneity of variance prior to being subjected to one-way ANOVA using Kolmogorov–Smirnov and Levene’s tests, respectively. Significant differences between means were determined by Tukey’s, Bonferroni and Holmes, or Scheffe’s multiple comparison tests, and the level of significance was set at *p* < 0.05 (* *p* < 0.05, ** *p* < 0.01, *** *p* < 0.001). Genes fold changes were submitted to a t-test (unpaired, parametric with Welch’s correction) with significance set at *p* < 0.05. All analyses were performed in GraphPad 9.5.0.

## 3. Results

### 3.1. Growth Performance

No significant differences were observed in the survival rate, the mean weight gain, the mean final weight, and the feeding rate (FR among the different groups (*p* < 0.05)). The specific growth rate (SGR) and feed conversion ratio (FCR) indicated significant differences (decreased and increased, respectively) among the various groups (Table 2). There was no mortality among the treatments.

### 3.2. Biochemical and Oxidative Stress Responses

**Figure 1:**
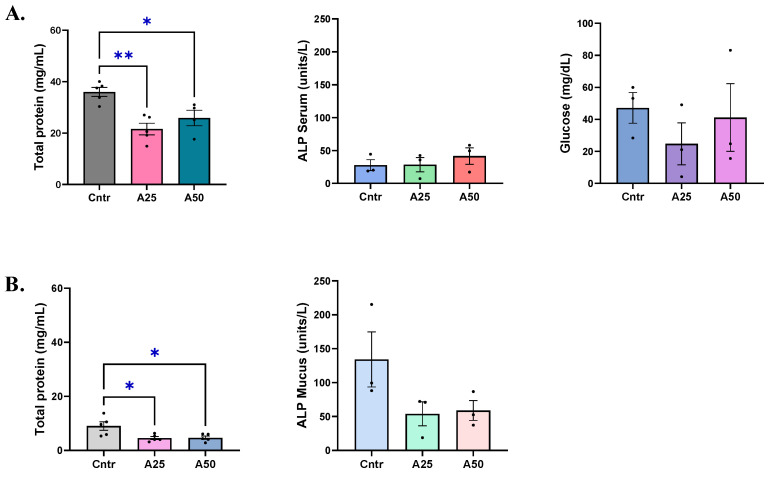
Total protein, alkaline phosphatase (ALP), and glucose levels in serum (**A**) and skin mucus (**B**) of *S. aurata* fed with *Artemisia arborescens* (AA) experimental diets. The total protein, alkaline phosphatase, and glucose levels are given as bar plots (mean value ± SEM; *n* = 3–5 fish per group). All statistical differences were assessed with one-way ANOVA followed by Tukey’s multiple comparison test. Significant differences between different diet groups and the control (Cntr) group are denoted by asterisks; *: *p* < 0.05, **: *p* < 0.01.

Serum total protein amounts were significantly altered by the A25 (21.6 mg mL^−1^) and the A50 (25.9 mg mL^−1^) experimental diets compared to the control diet (36 mg mL^−1^) (*p* = 0.029 and *p* = 0.028, respectively), but remained close to the control levels (Figure 1A). Skin-mucus total protein amounts were similar for both experimental diets (~4.6 mg mL^−1^), while the control group had 9 mg mL^−1^ of total proteins, indicating a significant decrease of skin-mucus total protein levels (*p* = 0.024 (A25); *p* = 0.027 (A50)) (Figure 1B). Alkaline phosphatase levels were stable in serum, ranging from 27.8 μL^−1^ to 28.5 μL^−1^ and 41.6 μL^−1^ for the control, A25, and A50 diets, respectively (Figure 1A). Skin-mucus alkaline phosphatase showed a decreasing trend in both experimental diets compared to the control diets (54 μL^−1^ (A25), 59 μL^−1^ (A50), and 134.2 μL^−1^ (control), respectively) (Figure 1B). Serum glucose levels appear to be slightly impacted by the experimental diets; however, no significant effects were observed (Figure 1A). 

**Figure 2:**
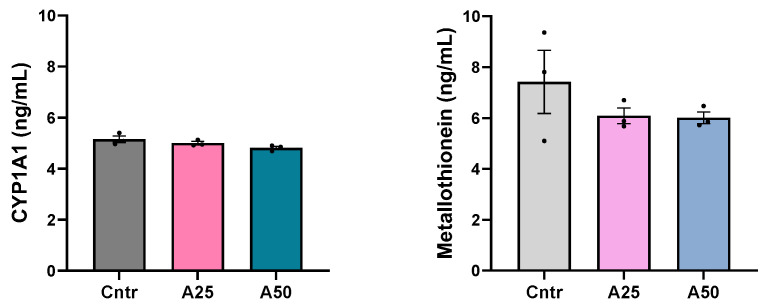
Cytochrome P450 1A1 (CYP1A1) and metallothionein levels in serum of *S. aurata* fed with *Artemisia arborescens* (AA) experimental diets. The CYP1A1 and metallothionein levels are given as bar plots (mean value ± SEM; *n* = 3 fish per group). All statistical differences were assessed with one-way ANOVA followed by Tukey’s multiple comparison test.

The cytochrome P450 1A1 concentration in serum was unaltered by the experimental diets (5, 4.8, and 5.2 ng mL^−1^ for A25, A50, and control diets, respectively), while serum metallothionein levels appear to be slightly lowered by the experimental diets (6.1, 6, and 7.4 ng mL^−1^ for A25, A50, and control diets, respectively) (Figure 2).

### 3.3. Non-Specific Immune Responses

**Figure 3:**
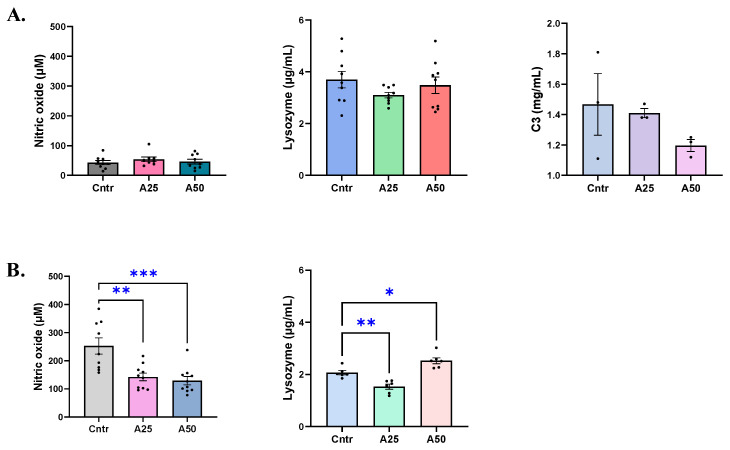
Nitric oxide, lysozyme, and complement C3 levels in serum (**A**) and skin mucus (**B**) of *S. aurata* fed with *Artemisia arborescens* (AA) experimental diets. The nitric oxide, lysozyme, and C3 levels are given as bar plots (mean value ± SEM; *n* = 3–10 fish per group). All statistical differences were assessed with one-way ANOVA followed by Tukey’s multiple comparison test. Significant differences between different diet groups and control (Cntr) group are denoted by asterisks; * *p* < 0.05, ** *p* < 0.01, *** *p* < 0.001.

Serum nitric oxide remained unaffected by the experimental diets, with nitric oxide levels ranging from 46.2 to 54 μM for the experimental and control diets (Figure 3A). On the contrary, a significant decrease in skin-mucus nitric oxide levels of fish fed A25 and A50 diets was observed when compared to the control diet (Figure 3B). The control diet had 252.5 µM of NO^−^ whereas A25 and A50 resulted in 142.4 and 129.1 µM NO^−^, respectively (*p* = 0.0015 (A25), *p* = 0.0004 (A50)). A similar trend was evident for lysozyme levels in serum and skin mucus. The experimental and control diets had similar serum lysozyme levels (Figure 3A), since the control lysozyme was 3.7 µg mL^−1^ while the A25 and A50 diets’ lysozyme ranged between 3.1–3.5 µg mL^−1^. The skin-mucus lysozyme of the A25 diets decreased significantly compared to the control diet (1.53 vs. 2.07 µg mL^−1^, respectively, *p* = 0.0045), while the A50 diet lysozyme was significantly increased (2.52 vs. 2.04, µg mL^−1^, respectively, *p* = 0.0138) (Figure 3B). The serum complement C3 levels were not affected by any of the experimental diets (1.4, 1.2, and 1.5 mg mL^−1^ for the A25, A50, and control diets, respectively) (Figure 3A).

**Figure 4:**
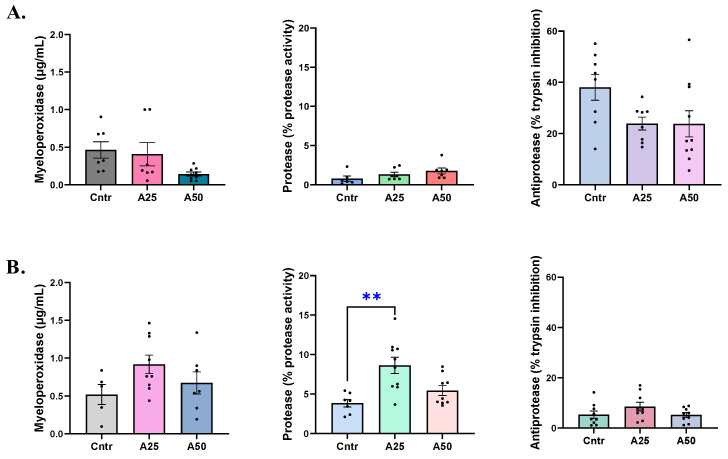
Myeloperoxidase, protease, and anti-protease activity levels in serum (**A**) and skin mucus (**B**) of *S. aurata* fed with *Artemisia arborescens* (AA) experimental diets. The myeloperoxidase, protease, and anti-protease activity levels are given as bar plots (mean value ± SEM; *n* = 5–10 fish per group). All statistical differences were assessed with one-way ANOVA followed by Tukey’s multiple comparison test. Significant differences between different diet groups and control (Cntr) group are denoted by asterisks; ** *p* < 0.01.

The serum myeloperoxidase levels were affected by the A50 experimental diet, as follows. The A50 MPO levels (0.14 µg mL^−1^) were lower than the A25 and control diet levels (0.41 and 0.46 µg mL^−1^, respectively, Figure 4A). The skin-mucus MPO levels increased in both experimental diets, having 0.92 µg mL^−1^ (A25) and 0.67 µg mL^−1^ (A50) of MPO compared to 0.52 µg mL^−1^ for the control diet (Figure 4B). The same trend was observed for skin-mucus protease activity levels. The A25 diet protease activity was significantly higher than the control diet (8.6 vs. 3.8% protease activity, respectively), and the A50 was also increased (5.4% protease activity) (Figure 4B). Serum protease activity, as well as serum and skin-mucus anti-protease activities, were not significantly affected by the experimental diets (Figure 4).

### 3.4. Immunoglobulin Response

**Figure 5:**
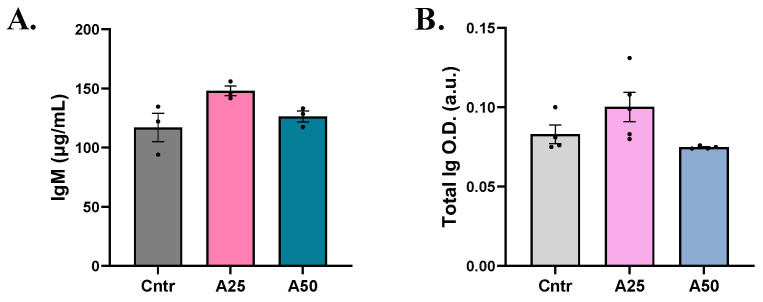
(**A**) Immunoglobulin M (IgM) and (**B**) total immunoglobulin levels in serum of *S. aurata* fed with *Artemisia arborescens* (AA) experimental diets. The Ig levels are given as bar plots (mean value ± SEM; *n* = 3–5 fish per group). All statistical differences were assessed with one-way ANOVA followed by Tukey’s multiple comparison test.

In serum, both total immunoglobulin and immunoglobulin M (IgM) levels were slightly increased with the A25 experimental diet compared to the control diet (total Ig: 0.1002 (A25) and 0.7475 (A50) a.u. vs. 0.083 a.u. (cntr); IgM: 148 (A25) and 126.3 (A50) µg mL^−1^ vs 116.9 µg mL^−1^ (cntr)). However, no significant modulation was observed (Figure 5).

### 3.5. Gene Expression

**Figure 6:**
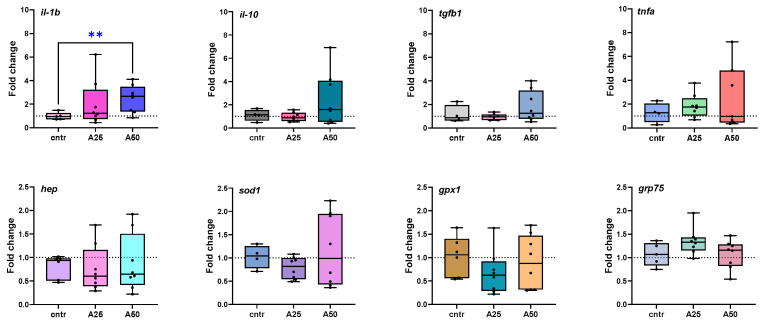
Fold-changes of differentially expressed genes in spleen tissue of *S. aurata* fed with *Artemisia arborescens* (AA) experimental diets. Fold changes are given as bounds of box and whisker plots with min-to-max values. The line represents the median (*n* = 5–9 fish per group), the whiskers show the data range, and the box shows the interquartile range. All statistical differences were assessed by t-test (unpaired, parametric with Welch’s correction). Significant differences between different diet groups and the control (Cntr) group are denoted by asterisks; ** *p* < 0.01.

All analyzed genes were differentially expressed to some extent and the corresponding fold changes (A25/cntr; A50/cntr) were graphically represented (Figure 6). In the spleen, the expression of interleukin-1 beta (*il-1b*) was slightly up-regulated in fish fed with A25, but it was significantly higher in fish fed with the A50 diet (*p* = 0.0078). The expression of interleukin-10 (*il-10*) tended to be up-regulated in fish fed with A25 but remained unaffected by the A50 diet. The same trend was observed for transforming growth factor beta 1 (*tgfb1*) and tumor necrosis factor alpha (*tnfa*) for both diets. Superoxide dismutase type 1 (*sod1*) and glutathione peroxidase 1 (*gpx1*) were slightly down-regulated by both experimental diets. Hepcidin (*hep*) gene expression was down-regulated for both experimental diets. Conversely, the 75-kDA glucose-regulated protein (*grp75*) was up-regulated for the group fed with A25 and essentially not altered when the fish were fed with an A50 diet. It should be noted that gene-expression differences were not statistically significant for any of the tested groups except (*il-1b*).

## 4. Discussion

In the present study, dietary supplementation with AA did not induce significant improvements in weight gain and seems to have a negative impact on the specific growth rate of the tested seabream specimens, despite the fact that the use of other *Artemisia* species has improved fish growth performance (e.g., *A. dracunculus* or tarragon [12]; *A. capillaries* [17,19], *A. absinthium* [9], or *A. annua* [13]). It has been suggested that the beneficial effects of plant supplementation on fish growth are dose-dependent, and if optimal doses are exceeded, the benefits might be lost [6]. Therefore, the observed effect on weight gain should be co-evaluated with biochemical and immune parameters taking into consideration the overall positive or negative outcome for *A. arborescens* evaluation on fish physiology.

Serum and plasma biochemical and immune parameters analysis is widely used for the assessment of the dietary effects of various substances on fish [36]. Therefore, the present study is focused on a wide range of biochemical parameters both in serum and mucus, in order to gain a more complete view of the fish status.

Blood contains extracellular proteins produced by the liver, suggesting a direct relationship between hepatic protein-synthesis levels and plasma total protein concentration. It has been reported that *Oncorhynchus mykiss* fed with 3% *Artemisia dracunculus* supplementation increased the concentration of total protein, possibly due to improved liver protein synthesis [12]. The same trend was observed for *A. annua* and *A. absinthium* feed supplementation in common carp and Nile tilapia [9,14,15]. In our study, AA caused a total protein decrease that was more intense for fish fed the A25 diet; thus, its effect on liver function should be further studied. Similarly, the skin-mucus total protein concentration appears to be slightly decreased in fish fed AA, an opposite trend of the use of *A. dracunculus* and *A. annua* [12,13].

Alkaline phosphatase (ALP) is a multifunctional membrane-bound enzyme that is involved in protein phosphorylation, cell growth, apoptosis, and cellular migration [37]. Moreover, mucus ALP exerts protective effects in the initial stage of wound healing, pathogen infection, or stress, due to hydrolytic activity [38]. Serum ALP is slightly affected by both AA experimental diets; however, mucus ALP is decreased. On the contrary, *A. dracunculus* supplementation resulted in a significant reduction in the activity of serum ALP and an increase in mucus ALP in *O. mykiss* [12].

Glucose is directly linked to carbohydrates and the stress hormone cortisol, which causes metabolic stress in fish, interfering with fish welfare [39]. *Oncorhynchus mykiss* fed with *A. dracunculus* and *Cyprinus carpio* fed with *A. annua* had significantly decreased glucose levels when compared to the basal diet group [12,14], a trend observed in the present study with AA diets, especially with the lower tested concentration. It has been found that phytochemical compounds of *A. dracunculus* have anti-diabetic properties [40]; therefore, the blood glucose reduction is anticipated. This observation is also supported by the increasing trend of glucose-regulated protein 75 (*grp75*) gene expression. Glucose-regulated protein 75 (GRP75/mortalin/mtHsp70/PBP74/HSPA9B) is a member of the heat-shock proteins 70 (Hsp70) family of chaperones and is implicated in multiple functions, ranging from stress response, intracellular trafficking, antigen processing, control of cell proliferation, differentiation, and tumorigenesis [41]. Specifically, (*grp75*) is triggered by glucose deprivation, oxidative injury, ionizing radiation, calcium ionophores, and hyperthyroidism [42].

Reactive oxygen species (ROS) play an important role in inflammatory disorders; however, they can be harmful at high levels. Antioxidant enzymes can defend against ROS excesses [16]; therefore, they are considered effective biomarkers for fish health-status evaluation. The detoxifying oxidative process that results in ROS formation can be divided into two phases. In phase I enzymes such as cytochrome P450 (CYP) enzymes convert compounds into more soluble compounds by oxidative reactions producing reactive products. In phase II, transferases catalyze the conjugation of endogenous molecules such as glutathione with the phase I end products in order to make them more soluble and less reactive. Antioxidant enzymes, such as superoxide dismutase (SOD) or glutathione peroxidase (GPx), are produced to counteract their effect [43]. SOD detoxifies the superoxide ion by decomposing it into hydrogen peroxide, which is the product that is decomposed to water and oxygen by catalase (CAT) and GPx [44]. Moreover, a positive correlation between antioxidant enzyme activity (CAT, SOD, GSH, and GPx) and innate immune response in fish has been proven [45]. It has been reported that *A. dracunculus* treatment resulted in a significant increase in the oxidative burst activity of rainbow trout blood leucocytes [12], and *A. absinthium* decreased oxidative stress (assessed by malondialdehyde (MDA) measurements). In that case, the authors observed that *A. absinthium* supplementation increased SOD and GPx, but decreased CAT activities, suggesting that such controversial results need more specific research [9]. Regarding the antioxidant gene-expression levels, *A. annua* and its mixture with *Foeniculum vulgare* significantly increased the mRNA expression of SOD and CAT in largemouth bass livers [16]. Similarly, *A. annua* treatments had significantly higher SOD, CAT, and GPx gene expression in common carp liver compared to the control group [44]. Several SOD, CAT, and GPx genes had significantly increased mRNA levels following the dietary addition of enzymatically treated *A. annua* on grass carp (*Ctenopharyngodon idella*) intestinal tissues [46]. *Sod* and *gpx* gene expression was also up-regulated when *A. cina* was added to catfish (*Clarias gariepinus*) feed [45]. In the present study, the *sod1* and *gpx1* expression levels seem stable compared to the control group, indicating that *A. arborescens* does not induce excessive oxidative stress in *S. aurata* spleen.

In an effort to tackle the effects of *A. arborescens* on the fish antioxidant mechanism from a different point of view, we studied CYP1A1 and metallothionein serum levels. Cytochrome P4501A (CYP1A) is among the major oxidative enzymes induced in fish by xenobiotics in a dose-dependent manner, with implications for toxicity, metabolism, and excretion of molecules, and has been studied in gilthead seabream [47]. The addition of *A. arborescens* in fish feed did not result in CYP1A1 production in serum; therefore, it can be assumed that oxidative stress is not induced by the supplement at least at a systemic level. Metallothionein (MT) is a low-molecular-weight metal-binding protein, which is implicated in fish protection against heavy metal toxicity, in homeostasis maintenance of essential trace elements such as zinc (Zn) and copper (Cu), and the cellular protection against oxidative stress [48]. Heavy metals (e.g., arsenic (As), chromium (Cr), cadmium (Cd), lead (Pb), and mercury (Hg)) accumulation can be harmless to fish and human health [49], and it has been reported that a low content of the toxic elements Cd and Pb were found in *Artemisia* tissue and infusion samples [11]. Therefore, MT was chosen not only as a marker to assess the oxidative effects of feed supplements but also whether the heavy metals *A. arborescence* contains can be toxic to gilthead seabream. Metallothionein levels in experimental fish serum appear to be lower compared to the control diet, indicating that *A. arborescence* use is relatively safe in that aspect.

Non-specific and immunoglobulin responses are direct biomarkers of the fish immune-system status, both in serum and mucus. Blood serum is a fairly labile biochemical system that reflects the condition of an organism in health and disease [50] while the fish skin mucus contains molecules that are responsible for immune defense, including lysozyme, complement, proteases, and immunoglobulins [51].

Lysozyme is one of the significant mucosal and humoral components of the immune system, protecting the fish against invasive microorganisms [36]. In the present study, serum lysozyme levels remain close to control-diet levels for both AA experimental feeds. However, a significant decrease of mucus lysozyme is recorded for the A25 diet, while in the A50 diet, its levels are significantly increased. The use of *A. dracunculus* led to a significant increase in serum and mucus lysozyme activity in rainbow trout [36]; feed supplementation with *A. afra* significantly increased lysozyme activity in *C. gariepinus* [21]. The use of *A. capillaries* induced the production of lysozyme in red seabream [18]. A. *absinthium and A. annua* increased lysozyme activity in common carp [9] and Nile tilapia [15], respectively, whereas findings for *A. annua’s* effects on the lysozyme levels in *C. carpio* were contradictory [13,14].

Fish complement can lyse foreign cells and opsonize foreign organisms for destruction by phagocytes [52]. Even though the use of *A. dracunculus* significantly increased the alternative complement activity [12], AA experimental diets resulted in a slight decrease in the C3 complement levels in *S. aurata*.

Protease enzymes play an important role in fish exerting a protective function against infectious diseases by cleaving microbial proteins and preventing their colonization. Also, the production of some other innate immune components in fish mucus (e.g., immunoglobulins, antibacterial peptides, or complement) could be activated and enhanced by proteases [36]. As expected, serum protease activities remain at low levels in serum for both experimental AA diets, similar to the control levels. However, mucus protease activity increases significantly in the mucus of fish fed with an A25 diet, suggesting that *A. arborescens* may increase mucus activity against pathogens, similar to the effects reported for *A. dracunculus* [12] and *A. annua* [13].

Since anti-proteases is a term used to characterize protease inhibitors, their production is expected to be low in mucus in order to maintain these high levels of mucus proteases active [36]. In the present study, the antiprotease activity is high in all fish groups’ serum (control and experimental) explaining the low protease activity levels described before.

To the best of our knowledge, neither nitric oxide nor myeloperoxidase have been previously studied for fish fed any *Artemisia* supplement. In fish, nitric oxide (NO) is involved in a large number of physiological processes, including development, cardiovascular homeostasis, neurotransmission, neuromodulation, and immune defense [53]. Gilthead seabream feeding with *A. arborescens* did not induce any change in serum nitric oxide levels; however, mucus NO levels were significantly lowered in both experimental groups compared to the control group. Only a few studies have assessed feed supplements’ effects on seabream nitric oxide levels, reporting a decrease in its serum levels by diatom *Phaeodactylum tricornutum* [54] or an increase of its mucus levels by the yeast *Kluyveromyces lactis M3* [55]; therefore, no direct comparison can be made.

Myeloperoxidase is a hemeprotein found in different leucocyte populations of invertebrate and vertebrate organisms, and it has been regarded as a marker of leucocyte activation in gilthead seabream [56]. Supplementation of feed with the yeast *Saccharomyces cerevisiae* enhanced the myeloperoxidase activity of seabream head kidney leucocytes [57]; however, in the present study, no significant difference was found for serum and mucus myeloperoxidase activity among the experimental diets.

Total immunoglobulins and IgM immunoglobulin are responsible for the production of antibody responses against antigens, contributing to fish resistance to pathogens [58]. In the present study, their levels were slightly increased, with low AA levels, and decreased with higher AA incorporation, in accordance with observations of *A. absinthium* and *A. annua* feed supplementation in common carp [9,13].

Only a few studies of Artemisia’s effects on various immune gene-expression levels were found. Addition of *A. cina* to catfish (*Clarias gariepinus*) feed down-regulated *tnfa* and *il-10* gene expression in the liver, intestine, and anterior kidney while *tgfb1* was significantly up-regulated in these tissues [45]. No significant difference in *tnfa* gene expression was observed with low supplementation levels of artemisin extracted from *A. carvifolia* in coral trout (*Plectropomus leopardus*) feed, contrary to high levels of artemisin which significantly down-regulated *tnfa* [59].

In the present study, genes related to cytokines (*il-1b*, *il-10*, *tgfb1*, *tnfa*, and *hep*) were analyzed in *S. aurata* spleen tissue. The fish spleen contains leukocytes, such as lymphocytes and macrophages, and its functions include hematopoiesis support, antibody production, erythrocytic destruction, blood filtration, and antigen degradation during an immune response [60]. It is also involved in tissue metabolism and oxidation [61], and it was chosen to be analyzed in order to obtain a more complete ‘snapshot’ of *A. arborescens* effect on *S. aurata*. Cytokines play a crucial role in fish health by regulating T and B lymphocytes-mediated immunity. Several essential cytokines, including tumor necrosis factor (TNFa), interleukins -1b and -10 (IL-1b, IL-10), and transforming growth factor-b (TGFb) have been identified in teleost fish [62]. IL-1b is a pro-inflammatory cytokine with various roles in inflammatory process regulation; it can increase phagocyte migration, macrophage activity, and lymphocyte activation [63]. *A. arborescens* significantly increases *il-1b* gene expression in the *S. aurata* spleen, a fact that can be interpreted either as a harmful effect of feed supplementation or as an immunostimulant effect of the feed supplementation. In general, the inflammatory response is a cascade triggered by TNF-a, which subsequently leads to the downstream transcription of other pro-inflammatory genes, including *il-1b* [64]. Fish TNF-α is mainly produced by macrophages and enhances the migration of immune cells [62]. TNF-a displays overlapping functions with IL-1b, which are tightly regulated, mainly at a transcriptional level, by anti-inflammatory cytokines. Since *A. arborescens* at a low level seems to slightly increase *tnfa* expression in the spleen but overall *tnfa* is not stimulated, it can be assumed that no inflammation is induced. Therefore, AA is not harmful to gilthead seabream.

Moreover, the anti-inflammatory cytokine IL-10 acts as a pro-inflammatory cytokine suppressor and exerts a conserved role in dampening inflammatory responses [63]. In fish, IL-10 significantly increases the number and enhances the phagocytic capacity of IgM^+^ B cells, promotes mature B-cell differentiation and proliferation, increases the number of IgM-secreting cells, and stimulates the secretion of antigen-specific IgM. IL-10 also could enhance the proliferation of T cells [62]. No *A. arborescens* effects are observed on *il-10* gene expression, supporting the hypothesis that no inflammation is induced in the *S. aurata* spleen. Finally, growth factors (GFs) are cytokines that stimulate cell proliferation, differentiation, and/or activation. TGF-b1 has an immunosuppressive function, regulates cell proliferation and viability, and suppresses erythropoiesis [62]. *tgfb1* gene expression on *S. aurata* spleen is not affected by *A. arborescens*; Therefore, it can be assumed that no immune or erythropoiesis occurs from AA feed supplementation.

Hepcidin (*hep*) is an antimicrobial peptide, and it plays a major role in innate immunity and iron regulation [65]. Hepcidin expression is strongly induced during infection and inflammation [66]. In fish, hepcidin has been proven to participate in the immune response [65]. Moreover, in humans, some foods and nutrients have a known or potential ability to influence hepcidin release [67], but the relationship between dietary changes in fish and hepcidin is not currently studied [66]. Hepcidin gene expression was found to be down-regulated by increasing phytic acid (PA) levels in the head kidney, spleen, and skin of grass carp (*Ctenopharyngodon idella*). PA is a common plant ingredient and a known anti-nutritional factor (ANF) for fish [68]. Moreover, dietary anti-nutrients (saponins and phytosterols) caused various hepcidin gene-expression-level modifications in gilthead seabream [69]. In the present study, both *A. arborescens* diets down-regulated hepcidin in the *S. aurata* spleen, as expected.

Overall, *A. arborescens* seems to induce the suppression of non-specific immune parameters in accordance with studies of plant-origin feed additives in other fish species [70]. Many studies, including those referenced for *Artemisia* species, report increased values of different immune parameters, and these results can be interpreted as inflammatory/hypersensitivity on the feed additive or immunostimulating effects of the supplementation [71]. In our opinion, the present results are built on the hypothesis that high replacement levels of fishmeal by plant ingredients could cause negative impacts on fish immune function [70,71]. Plants contain anti-nutritional factors (ANFs) for fish that result in their depressed immune function, as proven in a recent study on the head kidney, spleen, and skin of grass carp [68]. As mentioned above, dietary anti-nutrients (saponins (i.e., glycosides) and phytosterols) modulated *S. aurata* immune responses [69], and since *A. arborescens* contains a high amount of glycosides [11], it can be concluded that its use as a supplement in gilthead seabream feed should be further studied.

## 5. Conclusions

To the best of our knowledge, this is the first study of dietary *Artemisia arborescens* effects on *Sparus aurata*. A wide range of assays were used in order to have a more complete fish health-status assessment and nutritional changes effects. The spleen was chosen as the target tissue due to its basic role in immunity, as well as in metabolic and oxidation procedures. The vast majority of nutrition-related gene-expression studies are focused on liver or intestine function; therefore, spleen analysis offers an alternative point of view on plant-based diet on fish physiology. Our findings showed that *A. arborescens* use as a feed supplement could have a negative effect on the growth performance, immune response, and blood parameters of gilthead seabream. More studies should be conducted under experimental culturing conditions with more doses of *A. arborescens* to define a beneficial application range where the present findings may be reversed. Ideally, the optimal dose of *A. arborescens* must be assessed on seabream when it is subjected to stress conditions, including common pathogen infections. Therefore, our future goals include the study of *A. arborescens’* effects on gilthead seabream following well-designed experimental challenge tests with myxobacteria and parasites.

## Figures and Tables

**Table 1:** Formulation and proximate composition of the experimental diets supplemented with *Artemisia arborescens*.

Raw Materials	Control Diet (cntr)	Artemisia 0.25% (A25)	Artemisia 0.50% (A50)
Fish meal 67%	30.00	30.00	30.00
Soybean meal 45%	20.00	20.00	20.00
Wheat flour	14.38	14.38	14.38
Wheat gluten meal	6.00	6.00	6.00
Fish oil	13.24	12.99	12.74
Corn gluten meal	13.8	13.8	13.8
Monocalcium phosphate (MCP)	2.33	2.33	2.33
Vitamins and minerals premix	0.25	0.25	0.25
*Artemisia* E.O.	0	0.25	0.50
**Proximate Composition (%)**			
Crude Protein *	44.0	44.0	44.0
Crude lipid *	17.7	17.7	17.7
Carbohydrates **	1.8	1.8	1.8
Moisture *	-	-	-
Ash *	8.7	8.7	8.7
Starch ^†^	11.4	11.4	11.4
Phosphorus ^†^	1.4	1.4	1.4
Calcium ^†^	1.3	1.3	1.3
Lysine ^†^	2.4	2.4	2.4
Methionine + Cysteine ^†^	1.6	1.6	1.6
Gross Energy (MJ/kg) ***	20.8	20.8	20.8

* Samples of diets were analyzed for crude protein, total fat, moisture, and ash according to AOAC [26]. * Moisture content was measured after drying the samples at 105 °C for 24 h, ash was determined after ignition at 500 °C for 12 h, crude protein (N × 6.25) by the Kjeldahl method, and total fat was estimated gravimetrically by Soxhlet extraction. ** Calculated by difference: 100 − (%protein + %fat + %ash + %moisture) (i.e., N-free extractives + crude fiber). *** Gross energy was calculated using combustion values for protein 23.6 MJ kg^−1^*, lipid* 39.5 MJ kg^−1^, *and carbohydrate* 17.2 MJ kg^−1^*, respectively.* ^†^ Theoretical values predicted by Allix 3 formulation software.

**Table 2:** Growth-performance indices between groups.

Parameters	Control (cntr)	0.25% (A25)	0.5% (A50)	Significance
Mean Initial weight (g)	108.93 ± 4.21	109.94 ± 4.45	109.43 ± 2.53	p_1_ = 0.53/p_2_ = 0.84/p_3_ = 0.84
Mean Final weight (g)	208.00 ± 11.96	207.28 ± 14.31	201.91 ± 22.39	p_1_ = 0.24/p_2_ = 2.01/p_3_ = 1.76
Mean Weight gain (g)	99.06	97.34	92.47	N/A
FR (%)	1.5	1.5	1.5	N/A
SGR (%/day)	1.03 ± 0.04 ^a^	0.95 ± 0.02 ^b^	0.82 ± 0.02 ^c^	p_1_ = 0.029/p_2_ = 0.001/p_3_ = 0.006
FCR (%)	1.65 ± 0.03	1.82 ± 0.04	2.15 ± 0.13 ^a^	p_1_ = 0.093/p_2_ = 0.001/p_3_ = 0.008
Survival rate (%)	96.7	93.9	87.88	p_1_ = 0.83/p_2_ = 0.054/p_3_ = 0.111

Significant differences among groups are denoted with letters (^a,b,c^). p_1_: *p*-value of cntr vs. A25; p_2_: *p*-value of cntr vs. A50; p_3_: *p*-value of A25 vs. A50. All specific growth rates (SGRs) demonstrated significant differences among them. The A50 FCR exhibited a noteworthy distinction when compared to both the control and A25 FCRs.

## Data Availability

The data that support the findings of this study are available from the corresponding author, E.K., upon reasonable request. The data are not publicly available due to lack of appropriate qPCR and kits low-throughput raw data safe depository.

## Supplementary Materials

The following supporting information can be downloaded at: https://www.mdpi.com/article/10.3390/ani14081161/s1, Table S1: Primers and probes used in the present work.

## References

[1] FAO Food and Agriculture Organization of the United Nations, Blue Transformation. https://www.fao.org/fishery/en/bluetransformation.

[2] Stentiford G.D., Bateman I.J., Hinchliffe S.J., Bass D., Hartnell R., Santos E.M., Devlin M.J., Feist S.W., Taylor N.G.H., Verner-Jeffreys D.W. (2020). Sustainable aquaculture through the One Health lens. Nat. Food.

[3] Naylor R.L., Hardy R.W., Buschmann A.H., Bush S.R., Cao L., Klinger D.H., Little D.C., Lubchenco J., Shumway S.E., Troell M. (2021). A 20-year retrospective review of global aquaculture. Nature.

[4] Dawood M.A.O., Koshio S., Esteban M.Á. (2018). Beneficial roles of feed additives as immunostimulants in aquaculture: A review. Rev. Aquacult..

[5] Reverter M., Sarter S., Caruso D., Avarre J.C., Combe M., Pepey E., Pouyaud L., Vega-Heredía S., de Verdal H., Gozlan R.E. (2020). Aquaculture at the crossroads of global warming and antimicrobial resistance. Nat. Commun..

[6] Reverter M., Tapissier-Bontemps N., Sarter S., Sasal P., Caruso D. (2021). Moving towards more sustainable aquaculture practices: A meta-analysis on the potential of plant-enriched diets to improve fish growth, immunity and disease resistance. Rev. Aquacult..

[7] Aguiar G.A.C.C.d., Carneiro C.L.d.S., Campelo D.A.V., Rusth R.C.T., Maciel J.F.R., Baldisserotto B., Zuanon J.A.S., Oliveira A.V.d., Oliveira M.G.d.A., Freitas M.B.D.d. (2023). Effects of Dietary Peppermint (*Mentha piperita*) Essential Oil on Growth Performance, Plasma Biochemistry, Digestive Enzyme Activity, and Oxidative Stress Responses in Juvenile Nile Tilapia (*Oreochromis niloticus*). Fishes.

[8] Militello M., Settanni L., Aleo A., Mammina C., Moschetti G., Giammanco G.M., Blàzquez M.A., Carrubba A. (2011). Chemical composition and antibacterial potential of *Artemisia arborescens* L. essential oil. Curr. Microbiol..

[9] Yousefi M., Zahedi S., Reverter M., Adineh H., Hoseini S.M., Van Doan H., El-Haroun E.R., Hoseinifarm S.H. (2021). Enhanced growth performance, oxidative capacity and immune responses of common carp, *Cyprinus carpio* fed with *Artemisia absinthium* extract-supplemented diet. Aquaculture.

[10] Ekiert H., Klimek-Szczykutowicz M., Rzepiela A., Klin P., Szopa A. (2022). Artemisia Species with High Biological Values as a Potential Source of Medicinal and Cosmetic Raw Materials. Molecules.

[11] Lantzouraki D.Z., Amerikanou C., Karavoltsos S., Kafourou V., Sakellari A., Tagkouli D., Zoumpoulakis P., Makris D.P., Kalogeropoulos N., Kaliora A.C. (2023). *Artemisia arborescens* and *Artemisia inculta* from Crete; Secondary Metabolites, Trace Metals and in Vitro Antioxidant Activities. Life.

[12] Gholamhosseini A., Hosseinzadeh S., Soltanian S., Banaee M., Sureda A., Rakhshaninejad M., Ali Heidari A., Anbazpour H. (2021). Effect of dietary supplements of *Artemisia dracunculus* extract on the haemato-immunological and biochemical response, and growth performance of the rainbow trout (*Oncorhynchus mykiss*). Aquac. Res..

[13] Sarhadi I., Alizadeh E., Ahmadifar E., Adineh H., Dawood M.A. (2020). Skin mucosal, serum immunity and antioxidant capacity of common carp (*Cyprinus carpio*) fed artemisia (*Artemisia annua*). Ann. Anim. Sci..

[14] Hoseini S.M., Aydın B., Hoseinifar S.H., Moonmanee T., Van Doan H. (2022). Dietary Artemisia, *Artemisia annua*, supplementation improves common carp welfare under high stocking density. Aquac. Res..

[15] Soares M.P., Cardoso I.L., Ishikawa M.M., de Oliveira A.D.S.S., Sartoratto A., Jonsson C.M., de Queiroz S.C.D.N., Duarte M.C.T., Rantin F.T., Sampaio F.G. (2020). Effects of *Artemisia annua* alcohol extract on physiological and innate immunity of Nile tilapia (*Oreochromis niloticus*) to improve health status. Fish Shellfish Immunol..

[16] He G., Sun H., Liao R., Wei Y., Zhang T., Chen Y., Lin S. (2022). Effects of herbal extracts (*Foeniculum vulgare* and *Artemisia annua*) on growth, liver antioxidant capacity, intestinal morphology and microorganism of juvenile largemouth bass, *Micropterus salmoides*. Aquac. Rep..

[17] Ji S.C., Jeong G.S., Im G.S., Lee S.W., Yoo J.H., Takii K. (2007). Dietary medicinal herbs improve growth performance, fatty acid utilization, and stress recovery of Japanese flounder. Fish. Sci..

[18] Ji S.C., Takaoka O., Jeong G.S., Lee S.W., Ishimaru K., Seoka M., Takii K. (2007). Dietary medicinal herbs improve growth and some non-specific immunity of red sea bream *Pagrus major*. Fish. Sci..

[19] Takaoka O., Ji S.C., Ishimaru K., Lee S.W., Jeong G.S., Biswas A., Takii K. (2016). Dietary medicinal herbs and enzyme treated fish meal improve stress resistances and growth performance at early juvenile stage of red sea bream *Pagrus major*. Aquac. Res..

[20] Mbokane E.M., Moyo N.A.G. (2018). A preliminary investigation into the potential effect of *Artemisia afra* on growth and disease resistance in sub-adults of *Oreochromis mossambicus*. Aquaculture.

[21] Mbokane E.M., Moyo N.A.G. (2020). Effects of dietary levels of essential oil extracts from *Moringa oleifera* and *Artemisia afra* on kidney histology, haemato-immunological parameters and disease resistance in *Clarias gariepinus*. Aquac. Res..

[22] FAO (Food and Agriculture Organization of the United Nations) (2022). The State of Mediterranean and Black Sea Fisheries.

[23] FAO (Food and Agriculture Organization of the United Nations) (2022). The State of World Fisheries and Aquaculture 2022: Towards Blue Transformation.

[24] Mhalhel K., Levanti M., Abbate F., Laurà R., Guerrera M.C., Aragona M., Porcino C., Briglia M., Germanà A., Montalbano G. (2023). Review on Gilthead Seabream (*Sparus aurata*) Aquaculture: Life Cycle, Growth, Aquaculture Practices and Challenges. J. Mar. Sci. Eng..

[25] Mladineo I., Volpatti D., Beraldo P., Rigos G., Katharios P., Padros F. (2024). Monogenean *Sparicotyle chrysophrii*: The major pathogen of the Mediterranean gilthead seabream aquaculture. Rev. Aquac..

[26] Cunniff P.A., AOAC International (1995). Official Methods of Analysis of AOAC International.

[27] Wilkinson P.C. (1983). Methods for Studying Mononuclear Phagocytes. Immunology.

[28] Quade M.J., Roth J.A. (1997). A rapid, direct assay to measure degranulation of bovine neutrophil primary granules. Vet. Immunol. Immunopathol..

[29] Ross N.W., Firth K.J., Wang A., Burka J.F., Johnson S.C. (2000). Changes in hydrolytic enzyme activities of naïve Atlantic salmon *Salmo salar* skin mucus due to infection with the salmon louse *Lepeophtheirus salmonis* and cortisol implantation. Dis. Aquat. Organ..

[30] Henry M., Fountoulaki E. (2014). Optimal dietary protein/lipid ratio for improved immune status of a newly cultivated Mediterranean fish species, the shi drum *Umbrina cirrosa*. Fish Shellfish Immunol..

[31] Reyes-Becerril M., Salinas I., Cuesta A., Meseguer J., Tovar-Ramirez D., Ascencio-Valle F., Esteban M.A. (2008). Oral delivery of live yeast *Debaryomyces hansenii* modulates the main innate immune parameters and the expression of immune-relevant genes in the gilthead seabream (*Sparus aurata* L.). Fish Shellfish Immunol..

[32] Sitjà-Bobadilla A., Calduch-Giner J., Saera-Vila A., Palenzuela O., Alvarez-Pellitero P., Pérez-Sánchez J. (2008). Chronic exposure to the parasite *Enteromyxum leei* (Myxozoa: Myxosporea) modulates the immune response and the expression of growth, redox and immune relevant genes in gilthead sea bream, *Sparus aurata* L.. Fish Shellfish Immunol..

[33] Chaves-Pozo E., Liarte S., Fernández-Alacid L., Abellán E., Meseguer J., Mulero V., García-Ayala A. (2008). Pattern of expression of immune-relevant genes in the gonad of a teleost, the gilthead seabream (*Sparus aurata* L.). Mol. Immunol..

[34] Bustin S.A., Benes V., Garson J.A., Hellemans J., Huggett J., Kubista M., Mueller R., Nolan T., Pfaffl M.W., Shipley G.L. (2009). The MIQE guidelines: Minimum information for publication of quantitative real-time PCR experiments. Clin. Chem..

[35] Schmittgen T.D., Livak K.J. (2008). Analyzing real-time PCR data by the comparative C_(T)_ method. Nat. Protoc..

[36] Guardiola F.A., Cuesta A., Arizcun M., Meseguer J., Esteban M.A. (2014). Comparative skin mucus and serum humoral defence mechanisms in the teleost gilthead seabream (*Sparus aurata*). Fish Shellfish Immunol..

[37] Lallès J.P. (2019). Biology, environmental and nutritional modulation of skin mucus alkaline phosphatase in fish: A review. Fish Shellfish Immunol..

[38] Iger Y., Abraham M. (1990). The process of skin healing in experimentally wounded carp. J. Fish Biol..

[39] de Araujo F.C.T., Ribeiro R.P., Campos E.C., Todesco H., Tsujii K.M., Mantovani L.S.C., Ribeiro R.F., Carvalho J.C., Casetta J., Lopera-Barrero N.M. (2022). Could serum glucose be a selection criterion in Nile tilapia breeding programs?. Aquaculture.

[40] Eisenman S.W., Poulev A., Struwe L., Raskin I., Ribnicky D.M. (2011). Qualitative variation of anti-diabetic compounds in different tarragon (*Artemisia dracunculus* L.) cytotypes. Fitoterapia.

[41] Wadhwa R., Taira K., Kaul S.C. (2002). An Hsp70 family chaperone, mortalin/mthsp70/PBP74/Grp75: What, when, and where?. Cell Stress Chaperones.

[42] Bermejo-Nogales A., Benedito-Palos L., Saera-Vila A., Calduch-Giner J.A., Sitjà-Bobadilla A., Pérez-Sánchez J. (2008). Confinement exposure induces glucose regulated protein 75 (GRP75/mortalin/mtHsp70/PBP74/HSPA9B) in the hepatic tissue of gilthead sea bream (*Sparus aurata* L.). Comp. Biochem. Physiol. B Biochem. Mol. Biol..

[43] Capó X., Alomar C., Compa M., Sole M., Sanahuja I., Soliz Rojas D.L., González G.P., Garcinuño Martínez R.M., Deudero S. (2022). Quantification of differential tissue biomarker responses to microplastic ingestion and plasticizer bioaccumulation in aquaculture reared sea bream *Sparus aurata*. Environ. Res..

[44] Taheri Mirghaed A., Hamed Paknejad H., Mirzargar S.S. (2020). Hepatoprotective effects of dietary Artemisia (*Artemisia annua*) leaf extract on common carp (*Cyprinus carpio*) exposed to ambient ammonia. Aquaculture.

[45] El-Houseiny W., Anter R.G.A., Arisha A.H., Mansour A.T., Safhi F.A., Alwutayd K.M., Elshopakey G.E., Abd El-Hakim Y.M., Mohamed E.M.M. (2023). Growth Retardation, Oxidative Stress, Immunosuppression, and Inflammatory Disturbances Induced by Herbicide Exposure of Catfish, *Clarias gariepinus*, and the Alleviation Effect of Dietary Wormwood, *Artemisia cina*. Fishes.

[46] Dai Q.Q., Zhou X.Q., Jiang W.D., Wu P., Liu Y., Shi H.Q., Zhang L., Mi H.F., Tang J.Y., Zhang R.N. (2023). Application of enzymatically treated *Artemisia annua L.* on adult grass carp (*Ctenopharyngodon idella*): Improved growth performance, intestinal antioxidant capacity and apical junctional complex. Aquaculture.

[47] Ortiz-Delgado J.B., Segner H., Sarasquete C. (2005). Cellular distribution and induction of CYP1A following exposure of gilthead seabream, *Sparus aurata*, to waterborne and dietary benzo(a)pyrene and 2,3,7,8-tetrachlorodibenzo-p-dioxin: An immunohistochemical approach. Aquat. Toxicol..

[48] Guinot D., Ureña R., Pastor A., Varó I., del Ramo J., Torreblanca A. (2012). Long-term effect of temperature on bioaccumulation of dietary metals and metallothionein induction in *Sparus aurata*. Chemosphere.

[49] Tolkou A.K., Toubanaki D.K., Kyzas G.Z. (2023). Detection of Arsenic, Chromium, Cadmium, Lead, and Mercury in Fish: Effects on the Sustainable and Healthy Development of Aquatic Life and Human Consumers. Sustainability.

[50] Župan I., Tkalčić S., Čož-Rakovac R., Strunjak-Perović I., Topić-Popović N., Babić S., Bujak M., Šarić T. (2018). Biochemical parameters in the blood of gilthead sea bream (*Sparus aurata* Linnaeus, 1758) supplemented with commercially available β-glucan-based product (IMUNO-2865^®^). Aquac. Res..

[51] Subramanian S., MacKinnon S.L., Ross N.W. (2007). A comparative study on innate immune parameters in the epidermal mucus of various fish species. Comp. Biochem. Physiol. B Biochem. Mol. Biol..

[52] Holland M.C., Lambris J.D. (2002). The complement system in teleosts. Fish Shellfish Immunol..

[53] Locascio A., Annona G., Caccavale F., D’Aniello S., Agnisola C., Palumbo A. (2023). Nitric Oxide Function and Nitric Oxide Synthase Evolution in Aquatic Chordates. Int. J. Mol. Sci..

[54] Teixeira C., Peixoto D., Hinzmann M., Santos P., Ferreira I., Pereira G.V., Dias J., Costas B. (2022). Dietary Strategies to Modulate the Health Condition and Immune Responses in Gilthead Seabream (*Sparus aurata*) Juveniles Following Intestinal Inflammation. Animals.

[55] Guluarte C., Reyes-Becerril M., Gonzalez-Silvera D., Cuesta A., Angulo C., Esteban M.Á. (2019). Probiotic properties and fatty acid composition of the yeast *Kluyveromyces lactis* M3. In vivo immunomodulatory activities in gilthead seabream (*Sparus aurata*). Fish Shellfish Immunol..

[56] Chen Z., Ceballos-Francisco D., Guardiola F.A., Esteban M.Á. (2020). Influence of skin wounds on the intestinal inflammatory response and barrier function: Protective role of dietary *Shewanella putrefaciens* SpPdp11 administration to gilthead seabream (*Sparus aurata* L.). Fish Shellfish Immunol..

[57] Ortuño J., Cuesta A., Rodríguez A., Esteban M.A., Meseguer J. (2002). Oral administration of yeast, *Saccharomyces cerevisiae*, enhances the cellular innate immune response of gilthead seabream (*Sparus aurata* L.). Vet. Immunol. Immunopathol..

[58] Rombout J.H., Yang G., Kiron V. (2014). Adaptive immune responses at mucosal surfaces of teleost fish. Fish Shellfish Immunol..

[59] Lin Z.X., Pan L., Xie R.T., Li L.X., Wen J.S., Zhou X.Q., Dong X.H., Xie S.W., Tan B.P., Liu H.Y. (2023). Effects of dietary artemisinin on growth performance, digestive enzyme activity, intestinal microbiota, antioxidant capacity and immune biomarkers of Coral trout (*Plectropomus leopardus*). Aquac. Rep..

[60] Natnan M.E., Low C.F., Chong C.M., Bunawan H., Baharum S.N. (2021). Integration of Omics Tools for Understanding the Fish Immune Response Due to Microbial Challenge. Front. Mar. Sci..

[61] Zapata A.G. (2024). The fish spleen. Fish Shellfish Immunol..

[62] Cao J., Xu H., Yu Y., Xu Z. (2023). Regulatory roles of cytokines in T and B lymphocytes-mediated immunity in teleost fish. Dev. Comp. Immunol..

[63] Zou J., Secombes C.J. (2016). The Function of Fish Cytokines. Biology.

[64] Toubanaki D.K., Efstathiou A., Tzortzatos O.P., Valsamidis M.A., Papaharisis L., Bakopoulos V., Karagouni E. (2023). Nervous Necrosis Virus Modulation of European Sea Bass (*Dicentrarchus labrax*, L.) Immune Genes and Transcriptome towards Establishment of Virus Carrier State. Int. J. Mol. Sci..

[65] Cuesta A., Meseguer J., Esteban M.A. (2008). The antimicrobial peptide hepcidin exerts an important role in the innate immunity against bacteria in the bony fish gilthead seabream. Mol. Immunol..

[66] Shen Y., Zhao Z., Zhao J., Chen X., Cao M., Wu M. (2019). Expression and Functional Analysis of Hepcidin from Mandarin Fish (*Siniperca chuatsi*). Int. J. Mol. Sci..

[67] D’Andrea P., Giampieri F., Battino M. (2023). Nutritional Modulation of Hepcidin in the Treatment of Various Anemic States. Nutrients.

[68] Zhong J.R., Wu P., Feng L., Jiang W.D., Liu Y., Kuang S.Y., Tang L., Zhou X.Q. (2020). Dietary phytic acid weakened the antimicrobial activity and aggravated the inflammatory status of head kidney, spleen and skin in on-growing grass carp (*Ctenopharyngodon idella*). Fish Shellfish Immunol..

[69] Costas B., Couto A., Azeredo R., Machado M., Krogdahl A., Oliva-Teles A. (2014). Gilthead seabream (*Sparus aurata*) immune responses are modulated after feeding with purified antinutrients. Fish Shellfish Immunol..

[70] Burrells C., Williams P.D., Southgate P.J., Crampton V.O. (1999). Immunological, physiological and pathological responses of rainbow trout (*Oncorhynchus mykiss*) to increasing dietary concentrations of soybean proteins. Vet. Immunol. Immunopathol..

[71] Sitjà-Bobadilla A., Peña-Llopis S., Gómez-Requeni P., Médale F., Kaushik S., Pérez-Sánchez J. (2005). Effect of fish meal replacement by plant protein sources on non-specific defence mechanisms and oxidative stress in gilthead sea bream (*Sparus aurata*). Aquaculture.

