# Supplementary material for: Dietary Artemisia arborescens Supplementation Effects on Growth, Oxidative Status, and Immunity of Gilthead Seabream (Sparus aurata L.)
Source: Animals (Basel). 2024 Apr 11;14(8):1161. doi: 10.3390/ani14081161 (PMC11047518; doi:10.3390/ani14081161)
Supplement: Supplementary file 1 [file animals-14-01161-s001.zip › animals-2911934-supplementary.pdf]

animals

MDPI

Article

Dietary *Artemisia arborescens* Supplementation Effects on Growth, Oxidative Status, and Immunity of Gilthead Seabream (*Sparus aurata* L.)

Odysseas-Panagiotis Tzortzatos [1], Dimitra K. Toubanaki [1], Markos N. Kolygas [2], Yiannis Kotzamanis [3], Efstratios Roussos [3], Vasileios Bakopoulos [3], Achilleas Chatzopoulos [5], Fotini Athanassopoulou [2] and Evdokia Karagouni [1,*]

Supplementary material

Table S1. Primers and probes used in the present work.

Gene	Primer sequence (5′ - 3′)	Accession number	Product size (bp)	Reference
sod1	Up: TGTCTAACCTTGTCCGTTCTTC<br>Dp: CCGTTTATTCTACTTGTGCGTTAC	JQ308832.1	105	Present work
gpx1	Up: ATGGAAAAGATGCCCACCCC<br>Dp: CCTGACGGGACTCCAAATGA	DQ524992.1	116	Present work
hep	Up: GCCATCGTGCTCACCTTTAT<br>Dp: CTGCTGCCATACCCCATCTT	EF625900.1	152	[31]
grp75	Up: TCCGGTGTGGATCTGACCAAAGAC<br>Dp: TGTTTAGGCCCAGAAGCATCCATG	DQ524993.1	143	[32]
il-1b	Up: TCACTGGGCTGAACAACAG<br>Dp: GCACTCTCCTGGCACATATC	AJ277166.2	106	Present work
il-10	Up: TGCTCACTTGACTCTTCAGC<br>Dp: CAGGAGGAGAGACCGAGGAG	JX976621.1	114	Present work
tgfb	Up: CTCTCCTCAAACACACACAGAG<br>Dp: CCACATTTCTCTCGTCCTCTTC	AF424703.1	93	Present work
tnfa	Up: CCTCTCAGCCACAGGATCTC<br>Dp: CAGTTTGTCGCCTCTGTTCA	AJ413189.2	180	Present work
rsp18	Up: AGGGTGTTGGCAGACGTTAC<br>Dp: CTTCTGCCTGTTGAGGAACC	AM490061.1	164	[33]

References

31. Reyes-Becerril, M.; Salinas, I.; Cuesta, A.; Meseguer, J.; Tovar-Ramirez, D.; Ascencio-Valle, F.; Esteban, M.A. Oral delivery of live yeast *Debaryomyces hansenii* modulates the main innate immune parameters and the expression of immune-relevant genes in the gilthead seabream (*Sparus aurata L.*). *Fish Shellfish Immunol.* **2008**, *25(6)*, 731-739. https://doi.org/10.1016/j.fsi.2008.02.010.
32. Sitjà-Bobadilla, A.; Calduch-Giner, J.; Saera-Vila, A.; Palenzuela, O.; Alvarez-Pellitero, P.; Pérez-Sánchez, J. Chronic exposure to the parasite *Enteromyxum leei* (Myxozoa: Myxosporea) modulates the immune response and the expression of growth, redox and immune relevant genes in gilthead sea bream, *Sparus aurata L. Fish Shellfish Immunol.* **2008**, *24(5)*, 610-619. https://doi.org/10.1016/j.fsi.2008.01.014.
33. Chaves-Pozo, E.; Liarte, S.; Fernández-Alacid, L.; Abellán, E.; Meseguer, J.; Mulero, V.; García-Ayala, A. Pattern of expression of immune-relevant genes in the gonad of a teleost, the gilthead seabream (*Sparus aurata L.*). *Mol. Immunol.* **2008**, *45(10)*, 2998-3011. https://doi.org/10.1016/j.molimm.2008.01.018.

Disclaimer/Publisher's Note: The statements, opinions and data contained in all publications are solely those of the individual author(s) and contributor(s) and not of MDPI and/or the editor(s). MDPI and/or the editor(s) disclaim responsibility for any injury to people or property resulting from any ideas, methods, instructions or products referred to in the content.
